# Urinary Cysteinyl Leukotrienes as Biomarkers of Endothelial Activation, Inflammation and Oxidative Stress and Their Relationship with Organ Dysfunction in Human Septic Shock

**DOI:** 10.3390/biomedicines10112845

**Published:** 2022-11-08

**Authors:** Marta Reina-Couto, Marisa Santos-Oliveira, Patrícia Pereira-Terra, Carolina Silva-Pereira, Janete Quelhas-Santos, Álvaro Duarte, Sandra Martins, Paula Serrão, Cláudia Camila Dias, Manuela Morato, João T. Guimarães, Roberto Roncon-Albuquerque, José-Artur Paiva, António Albino-Teixeira, Teresa Sousa

**Affiliations:** 1 Departamento de Biomedicina—Unidade de Farmacologia e Terapêutica, Faculdade de Medicina da Universidade do Porto, 4200-319 Porto, Portugal; 2 Centro de Investigação Farmacológica e Inovação Medicamentosa, Universidade do Porto (MedInUP), 4200-319 Porto, Portugal; 3 Serviço de Medicina Intensiva, Centro Hospitalar Universitário São João, 4200-319 Porto, Portugal; 4 Serviço de Farmacologia Clínica, Centro Hospitalar Universitário São João, 4200-319 Porto, Portugal; 5 Serviço de Patologia Clínica, Centro Hospitalar Universitário São João, 4200-319 Porto, Portugal; 6 EPIUnit, Instituto de Saúde Pública, Universidade do Porto, 4050-600 Porto, Portugal; 7 Departamento de Medicina da Comunidade, Informação e Decisão em Saúde, Faculdade de Medicina da Universidade do Porto, 4200-319 Porto, Portugal; 8 Centro de Investigação em Tecnologias e Serviços de Saúde (CINTESI), Universidade do Porto, 4200-450 Porto, Portugal; 9 Laboratório de Farmacologia, Departamento de Ciências do Medicamento, Faculdade de Farmácia da Universidade do Porto, 4050-313 Porto, Portugal; 10 LAQV/REQUIMTE, Faculdade de Farmácia da Universidade do Porto, 4050-313 Porto, Portugal; 11 Departamento de Bioquímica, Faculdade de Medicina da Universidade do Porto, 4200-319 Porto, Portugal; 12 Departamento de Cirurgia e Fisiologia, Faculdade de Medicina da Universidade do Porto, 4200-319 Porto, Portugal; 13 Departamento de Medicina, Faculdade de Medicina da Universidade do Porto, 4200-319 Porto, Portugal

**Keywords:** cysteinyl leukotrienes, septic shock, endothelial activation, inflammation, oxidative stress, organ dysfunction

## Abstract

Cysteinyl leukotrienes (CysLT) are potent vascular leakage-promoting agents but have been scarcely explored in human septic shock (SS). We evaluated CysLT at admission and during hospitalization and their correlation with endothelial dysfunction, inflammation, oxidative stress, the renin–angiotensin–aldosterone system, and cardiac, renal, respiratory, and hepatic parameters in SS patients. Blood and spot-urine samples were collected at days 1–2 (admission), 3–4, and 5–8 in SS patients (*n* = 13) and at a single time point in controls (*n* = 22). Urinary CysLT (u-CysLT) and isoprostanes, plasma, and urinary angiotensinogen, serum myeloperoxidase, and IL-10 were quantified by ELISA. Serum intercellular-adhesion molecule-1, vascular cell-adhesion molecule-1, E-selectin, tumor necrosis factor-α, IL-1β, and IL-6 were measured by multiplex immunoassays. Routine markers were evaluated using automated analyzers. At admission, SS patients had increased u-CysLT, endothelial activation, inflammation, oxidative stress, and plasma and urinary angiotensinogen, as well as cardiac, respiratory, hepatic, and renal injury/dysfunction. There were no changes in u-CysLT during hospitalization. Both correlation and multivariate analyses showed positive relationships of u-CysLT with endothelial activation, inflammation, oxidative stress, proteinuria, and hepatic injury/dysfunction markers. These results suggest that u-CysLT may be potential non-invasive biomarkers for monitoring the pathophysiological mechanisms underlying SS, as well as putative therapeutic targets.

## 1. Introduction

The Third International Consensus Definitions for Sepsis and Septic Shock [1] established recently that “sepsis is defined as life-threatening organ dysfunction caused by a dysregulated host response to infection”. This new definition features a multifaceted pro- and anti-inflammatory host response to an infecting pathogen and evidences the biological and clinical heterogeneity of the affected individuals [2,3]. In addition, focusing on multiorgan failure as an adaptive response [4,5] allows its operationalization (acute change in total Sequential Organ Failure Assessment (SOFA) score ≥2 points consequent to the infection) to be a potentially reversible process [6]. Septic shock (SS), a subset of sepsis in which underlying circulatory and cell-metabolic abnormalities are profound enough to substantially increase mortality [7], can be identified with a clinical construct of sepsis with persisting hypotension requiring vasopressors to maintain a mean arterial pressure (MAP) of 65 mm Hg and having a serum lactate level >2 mmol/L (18 mg/dL) despite adequate volume resuscitation [8]. Of the nearly 250 biomarkers evaluated for sepsis, none is yet routinely applied in clinical management, and one of the major challenges in the near future will be to validate biomarkers that can be easily measured and yet reflect pathophysiological events, thus allowing the development of personalized therapeutics [9]. Although procalcitonin (PCT) and C-reactive protein (CRP) are the most frequently used in clinical practice, the most recent Surviving Sepsis Campaign guidelines for the management of sepsis mention that sepsis biomarkers, specifically PCT [10], are not superior to clinical evaluation [11]. Except for recommended use of lactate both in the Guidelines and in the Sepsis-3 definition consensus, the role of biomarkers in sepsis diagnosis remains poorly undefined [7].

Both sepsis and SS are widely recognized as one of the main factors contributing to high mortality and morbidity in hospital settings [12,13], with mortality rates being around 20–30% in SS [14,15,16]. The identification of the mediators involved in the pathophysiology of sepsis is imperative for future development of novel therapeutics [7,17]. This is particularly relevant since current treatment of septic patients consists mainly of limited specific (antibiotics and source control) and general supportive measures [18]. Nowadays, the pathophysiology of sepsis is viewed as a dysregulated homeostasis arising not only from sustained excessive inflammation but also from immune suppression [19]. The failure of anti-inflammatory-agent trials, including corticosteroids [20], tumor necrosis factor (TNF) antagonists [21], interleukin-1 (IL-1)–receptor antagonists [22], and other anti-inflammatory drugs [23], questions the theory of uncontrolled inflammation [24].

Endothelial damage is one of the SS hallmarks closely interconnected with inflammatory response. It is characterized by a phenotypic switch of the endothelial cells toward a proadhesive, proinflammatory, proapoptotic, procoagulant and prooxidant state, loss of barrier function, and vascular leakage, which contribute to diffuse tissue edema and vasodilation [25,26]. These changes ultimately result in hypotension, inadequate organ perfusion [26] with combined breakdown of both the endothelial and epithelial barriers, and further widespread lethal organ dysfunction [4]. The resulting increased fluid in the interstitial space is associated with a higher mortality rate in these patients [26]. 

Leukotrienes (LT) are becoming targets of increasing attention since the available evidence outlines them as major endogenous mediators responsible for the development of sepsis [27,28,29], potentially by leukocyte recruitment, adhesion of leukocytes to endothelial cells, induction of inflammatory cytokine production, vascular endothelial damage, and vascular leakage [30]. The generation of LTs mainly occurs in immune-defense cells, such as macrophages, monocytes, neutrophils, eosinophils, mast cells, and basophils [31]. This process initiates with the cleavage of arachidonic acid (AA) from the lipid-cell membrane by the action of cytosolic phospholipase A_2_ [32]. Upon AA release, the 5-lipoxygenase (5-LO)-activating protein (FLAP) presents AA to 5-LO, which converts it to 5-hydroperoxyeicosatetraenoic acid (5-HPETE) and then to LTA_4_, an unstable epoxide [33]. Further LTA_4_ metabolism is local-dependent: in neutrophils, LTA_4_ hydrolase generates LTB_4_ [34], but in other immune cells, LTC_4_ synthase (LTC_4_S) produces LTC_4_, the first cysteinyl LT (CysLT) in the pathway [35]. LTC_4_ is transported to the extracellular space and sequentially cleaved by γ-glutamyl transpeptidase and dipeptidases producing LTD_4_ and LTE_4_, respectively [36,37]. Once in the blood circulation, LTC_4_ and LTD_4_ are rapidly converted into LTE_4_, which has a short half-life of up to 4 min [38], thus making blood samples obsolete for the quantification of these molecules [39]. On the other hand, urinary excretion of LTE_4_ is a good indicator of short-term variations in the synthesis of LTC_4_, with long-term changes being reflected by ω- and β-oxidation metabolites of LTE_4_ [40]. Therefore, urine appears to the best biological matrix for the measurement of CysLT metabolites [39]. Of note, the transcellular biosynthesis of CysLT also occurs since cells unable to produce LTA_4_, including vascular endothelial cells, platelets, human airway epithelial cells, alveolar macrophages, blood-peripheral monocytes, and kidney-derived endothelial cells, can use the LTA_4_ generated by neutrophils or other cells in the surrounding tissues to produce CysLT. Under inflammatory conditions, this process may elicit high local amounts of these mediators [41].

CysLT_1_ receptor and CysLT_2_ receptor are two identified receptors for CysLT mediating their biological effects. These receptors have different expression sites and binding affinity for CysLT, which may suggest different functions in vivo [42,43]. In addition to these classical receptors, CysLT also appear to bind to other G-protein coupled receptors (GPCR or GPR), namely, the orphan receptors, GPR17 and GPR99. GPR17 has been described as a negative regulator of the CysLT_1_ receptor response to LTD_4_ [44], and GPR99 is a newer receptor proposed as a probable LTE_4_ receptor, since it can mediate LTE_4_ oedema responses, even when CysLT_1_ and CysLT_2_ receptors are absent [45].

Despite their multiple proinflammatory actions, evidence of the pathophysiological role of CysLT in human sepsis is still scarce [46]. Due to the ability of these mediators to increase microvasculature permeability [42,47,48,49] and participate in innate and adaptive immune responses [50], their involvement in the development of septic endothelial injury is likely conceivable. Indeed, several studies in experimental models of sepsis have highlighted CysLT as important mediators of sepsis pathogenesis and progression [51,52,53,54,55]. Importantly, the ex vivo generation of the CysLT LTC_4_ was already demonstrated to be impaired in sepsis and suggested as a biomarker for survival in critically ill patients [46].

As previously mentioned, there has been a failure of targeted therapies to modify the natural course of sepsis [19]. Given the heavy burden of this condition, new disease-modifying therapies are in demand. Of note, treatment with montelukast, an antagonist of CysLT_1_ receptor, has shown promising results in experimental models of sepsis by inhibiting the recruitment of neutrophils, decreasing reactive oxygen species (ROS) and inflammatory cytokines, and consequently protecting against oxidative stress-induced organ dysfunction [53,54,55].

In the present study, we aimed to quantify urinary CysLT in SS patients at admission and during hospitalization. We also assessed the relationship of CysLT with biomarkers of endothelial activation, inflammation, oxidative stress, renin–angiotensin–aldosterone system (RAAS) activation, and organ dysfunction in order to identify the pathophysiological processes associated with these mediators and evaluate their utility as biomarkers for the clinical management of SS patients, as well as their relevance as therapeutic targets to explore in the future.

## 2. Materials and Methods

### 2.1. Study Subjects

The present study is part of a larger research project (RIFF-HEART) involving patients from the Intensive Care Unit (ICU) of the Service of Intensive Care Medicine of a tertiary hospital with the diagnosis of acute heart failure (AHF), cardiogenic shock (CS), and non-cardiogenic shock (septic shock, SS).

We performed a single-center cohort study and recruited patients with the diagnosis of SS (*n* = 20), admitted to the ICU of the Service of Intensive Care Medicine of Centro Hospitalar e Universitário São João (CHUSJ), from January 2017 to December 2019. Controls (*n* = 22) were recruited among healthy blood-donor volunteers from the Service of Immunohemotherapy of CHUSJ from September 2017 to October 2017. Patients were enrolled if they met all inclusion criteria: (1) evidence of infection, (2) evidence of ≥ 1 organ dysfunction identified as an acute change in total SOFA score ≥2 points consequent to the infection with a cardiovascular SOFA of at least ≥2 points and a serum-lactate level > 2 mmol/L [1], and (3) evidence of organ dysfunction present for no longer than 48 h. Patients who had an acute cerebral vascular event, acute coronary syndrome, acute pulmonary edema, status asthmaticus, active cardiac arrhythmias, active gastrointestinal hemorrhage, pregnancy, seizure, drug overdose, burn injury, trauma, requirement for immediate surgery, or advanced-stage cancer were excluded. Other exclusion criteria were being younger than 18 years or having a history of glucocorticoid or other immunosuppressive medication. All eligible patients (or their legal representative) provided written informed consent to participate in the study. Blood-donor volunteers provided oral informed consent. For analysis purposes, we only included the participants in whom it was possible to collect both blood and spot-urine samples at admission. Since in the SS group there were 7 patients without a urine sample at admission, they were excluded from the analyses. Therefore, we had a final number of 13 SS patients at admission. The study was conducted in accordance with the Guidelines for Good Clinical Practice and the 1975 Declaration of Helsinki, after approval by the institution’s ethics committee (CES 75-16). Patients were followed during their stay in the ICU by the medical team of the project. 

### 2.2. Clinical Data and Sample Collection

Illness severity was assessed by ICU scoring systems, namely, the Acute Physiology and Chronic Health Evaluation II (APACHE II), the Simplified Acute Physiology Score II (SAPS II), and the SOFA score at the time of ICU admission. The SOFA score was also evaluated throughout hospitalization. Physical examination of the patients was performed throughout their ICU stay and a record of demographic and clinical data for each patient was completed by the medical team and further anonymously coded to the project database, along with laboratory data, guaranteeing confidentiality. 

For SS patients, blood and spot-urine samples were collected at 3 different time points throughout their hospital stay, whenever possible: up to 48 h (days 1–2; admission), on days 3–4, and on days 5–8 after ICU admission. Samples (blood and spot urine) from controls were collected at a single time point. All samples were processed within 1–2 h of collection and stored at −80 °C until assayed. 

### 2.3. Routine Laboratory Procedures

Most routine laboratory analyses were performed at the Service of Clinical Pathology of CHUSJ. Quantification of plasma B-type natriuretic peptide (BNP) and high-sensitivity troponin I (hsTnI) was performed by chemiluminescent microparticle immunoassays using an Abbot^®^ Architect i2000 automated analyzer (Abbott^®^ Diagnostics, Lake Forest, IL, USA) and respective Abbot reagents (ARCHITECT BNP assay and ARCHITECT STAT High Sensitivity Troponin-I, Abbott^®^ Diagnostics, Lake Forest, IL, USA). 

A Beckman Coulter^®^ AU5400 automated clinical chemistry analyzer (Beckman Coulter^®^, Portugal) was used for the quantification of serum CRP (s-CRP) by an immunoturbidimetric assay, serum urea concentration by a kinetic urease/glutamate dehydrogenase method, aspartate aminotransferase (AST) and alanine aminotransferase (ALT) by kinetic photometric assays, gamma glutamyltransferase (GGT) by a kinetic colorimetric method, alkaline phosphatase (ALP) and total and direct bilirubin by colorimetric assays, plasma and urine creatinine by the colorimetric Jaffe method, and urinary protein by the pyrogallol red method. All the reagents used for these assays were from Beckman Coulter Ireland Inc., Lismeehan, O’Callaghan’s Mill’s, Co. Clare, Ireland. 

Differential leukocyte count was analyzed by flow cytometry in an automated hematology-analysis system (Sysmex 5000; Emılio de Azevedo Campos, Porto, Portugal) and prothrombin time (PT) was performed by a coagulometric method using the Thromborel^®^ S reagent (Siemens Healthcare Diagnostics, Tarrytown, NY, USA) in an automated analyzer (STA R Max^3^; Stago, Amadora, Portugal) at the Service of Immunohemotherapy, CHUSJ. 

These routine clinical markers/parameters were measured only in SS patients, with the exception of plasma and urine creatinine, which were also evaluated in controls.

### 2.4. Quantification of Urinary Cysteinyl Leukotrienes

CysLT were measured in unextracted spot-urine (u-) samples (u-CysLT) by a competitive enzyme-linked immunosorbent assay (ELISA) using a commercial kit (Cysteinyl Leukotriene ELISA kit, Cayman Chemical Company, Ann Arbor, MI, USA). The concentrations of u-CysLT (pg/mL) were further divided by the respective urinary creatinine values (mg/mL) to account for spot-urine dilution and were expressed in pg/mg creatinine.

### 2.5. Quantification of Urinary Isoprostanes

Urinary isoprostanes (u-Isop) were measured by a competitive enzyme immunoassay (Urinary Isoprostane ELISA Kit; Oxford Biomedical Research^®^ Inc., Oxford, MI, USA) in non-extracted spot urine containing the preservative butylated hydroxy toluene (BHT, 0.005%, *w*/*v*) added before storage. The values of u-Isop (ng/mL) were further divided by the respective urinary creatinine content (mg/mL) to account for spot-urine dilution and were expressed in ng/mg creatinine.

### 2.6. Measurement of Serum Endothelial Activation and Inflammatory Biomarkers

Serum (s-) endothelium-activation markers (intercellular-adhesion molecule 1, s-ICAM-1; vascular cell-adhesion molecule 1, s-VCAM-1; E-Selectin, s-E-Selectin) and proinflammatory cytokines (tumor necrosis factor alpha, s-TNF-α; interleukin-1 beta, s-IL-1β; interleukin-6, s-IL-6) were evaluated by multiplex immunoassays using a Luminex 200TM xMAP^TM^ analyzer (Luminex Corporation, Austin, TX, USA), according to the protocols of Human Premixed Multi-Analyte Magnetic Assay (R&D Systems, Inc., Minneapolis, MN, USA) and Human High Sensitivity T Cell Magnetic Bead Panel (Milliplex^®^ Map kit, Millipore Corporation, Billerica, MA, USA), respectively. Raw-data analysis (mean fluorescence intensity) was performed using a standard five-parameter-logistic (5-PL) curve fit created by the Luminex xPONENT^®^ Software (version 3.1). Serum interleukin-10 (s-IL-10) and serum myeloperoxidase (s-MPO) were quantified by enzyme immunoassays using commercial kits (Thermo Scientific^TM^ Human Interleukin-10 (IL-10) ELISA Kit, Thermo Scientific, Rockford, IL, USA; BioCheck MPO Enzyme Immunoassay, Oxis International Inc., Foster City, CA, USA).

### 2.7. Quantification of Plasma and Urinary Angiotensinogen

Angiotensinogen was measured in plasma (p-AGT) and spot-urine (u-AGT) samples using an enzyme-linked immunosorbent-assay test (Human Total Angiotensinogen Assay Kit, Immuno-Biological Laboratories Co., Gunma, Japan), following the instructions provided by the manufacturer. The values of u-AGT values (ng/mL) were further divided by the respective urinary creatinine concentrations (mg/mL) to account for spot-urine dilution and were expressed in ng/mg creatinine.

### 2.8. Data and Statistical Analysis

Results are expressed as mean ± standard error of the mean (SEM) or as median (25th percentile; 75th percentile) or percentage and are graphically represented by box-and-whisker plots and scatter plots. The estimated glomerular-filtration rate (eGFR) was calculated using the Chronic Kidney Disease Epidemiology Collaboration (CKD-EPI) equation [56]. Statistical analysis for the comparison of values on admission was performed using the unpaired Student’s *t*-test or Mann–Whitney U-test, where appropriate. Categorical variables were analyzed by the Fisher’s exact test. Evolution of biomarkers or parameters throughout the hospitalization was analyzed by Wilcoxon matched-pairs signed-rank test or by paired *t*-test, where appropriate. Spearman’s correlation was used to analyze correlations between nonparametric data in the study population (both in the entire study population at admission and separately in controls and SS patients at admission). Repeated-measure multivariate analyses were conducted to determine the relationship between u-CysLT and endothelial-activation markers, inflammation and oxidative stress markers, markers of systemic/intrarenal RAAS and renal injury, and hepatic markers throughout hospitalization, adjusted for some confounders, namely, age, gender, and eGFR. The coefficient of regression (β), 95% confidence intervals (95% CI), and *p*-value were presented. A *p*-value of <0.05 was considered significant. Statistical analysis was performed using the GraphPad Prism 9 software (La Jolla, CA, USA) and the IBM SPSS Statistics 27 software (IBM Corporation, New York, NY, USA). To prevent possible bias in clinical evaluation, all the patients were examined by the same medical team included in the project. However, we cannot exclude bias from eventual patient selection because most patients recruited had urosepsis to meet the demanding inclusion criteria. To assure comparability of the biomarker assessment, samples from controls and SS patients were evenly distributed in each assay plate. There were missing values in some biomarkers due to insufficient volume to perform sample processing, dilution tests, and assays. Furthermore, in some of the 13 SS patients included in this study, it was not possible to collect blood and/or urine samples at all time points during hospitalization due to patient discharge from the ICU, ICU team logistics, or the lack of a sufficient urine excretion (in the case of urine collection). For the follow-up after ICU admission, we included and analyzed patients that had at least one blood/urine sample collected at days 3–4 or at days 5–8. The final number of patients with blood and urine samples at days 3–4 was 8 and 7, respectively, and for days 5–8 was 6 and 5, respectively. In addition, some routine clinical biomarkers could only be assessed at a single time point during hospitalization due to the hospital’s internal policies, and we did not have permission to measure routine clinical biomarkers in controls (blood-donor volunteers), with the exception of creatinine, or have access to their hospital laboratory reports. The final number per group for the biomarkers/parameters evaluated at admission was as follows: lactate, *n* = 13 (SS); P/F, *n* = 12 (SS); APACHE II, SAPS II, SOFA, *n* = 13 (SS); p-BNP, *n* = 8 (SS); p-hsTnI, *n* = 13 (SS); eGFR, *n* = 13 vs. 22 (SS vs. controls); urea, *n* = 13 (SS); u-protein/creatinine ratio, *n* = 10 (SS); AST, ALT, ALP, GGT, total bilirubin, *n* = 13 (SS); PT, *n* = 12 (SS); u-CysLT, *n* = 13 vs. 22 (SS vs. controls); s-ICAM-1, s-VCAM-1, s-E-Selectin, s-TNF-α, s-IL-1β, s-IL-6, s-MPO, *n* = 13 vs. 22 (SS vs. controls); s-IL-10, *n* = 12 vs. 22 (SS vs. controls); s-CRP, total leukocyte count, neutrophils, monocytes, lymphocytes, NLR, NMR, *n* = 13 (SS); u-Isop, *n* = 13 vs. 22 (SS vs. controls), p-AGT, *n* = 13 vs. 22; u-AGT, *n* = 13 vs. 22 (SS vs. controls). To avoid biasing the results, no imputation for missing values was used. Sample size was defined according to the RIFF-HEART project’s primary objectives, which consisted of characterizing resolution of inflammation and endothelitis at admission and during hospitalization. Reporting of the study conforms to the STROBE statement along with references to STROBE and the broader EQUATOR guidelines [57].

## 3. Results

### 3.1. Demographic, Clinical, and Biochemical Characteristics

Demographic, clinical, and biochemical characteristics of the subjects included in the study are presented in Table 1.

**Figure 1:**
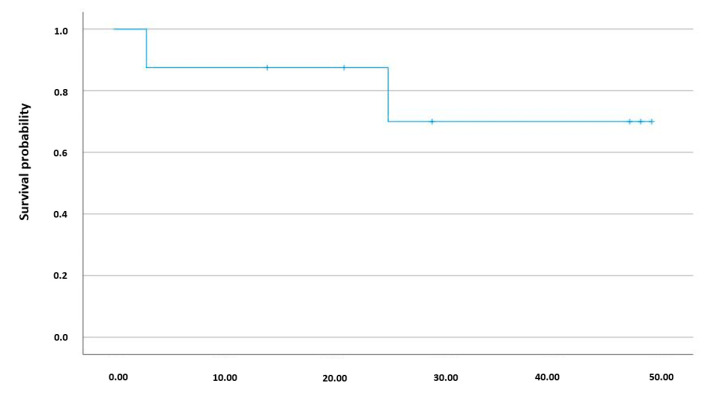
Kaplan–Meier survival plot: Kaplan–Meier analysis showing the survival rate among the 13 SS patients for a period of 48 months after hospital admission.

There were 22 controls and 13 SS patients that did not significantly differ in age and gender. SS patients presented moderately to severely impaired renal function (eGFR (mL/min per 1.73 m^2^): 36 ± 6)), with 2 patients requiring renal replacement therapy. SS patients also had acute hypoxemic respiratory failure (P/F < 300), with one patient requiring VV-ECMO. Patients with SS exhibited raised values of cardiac and some hepatic biomarkers (AST, PT), indicating heart and liver damage. Cardiovascular and metabolic diseases were the most common comorbidities. Renal and genitourinary tract infection was the predominant source of sepsis, with 54% of the patients presenting a Gram-negative infection. Regarding therapeutics at admission, the first empiric antibiotic used was ceftriaxone in 69% of the patients and piperacillin/tazobactan in 31%, whereas 31% of them were put on dual or triple antibiotic association and 1% of the patients also received antiviral therapy. The mean norepinephrine dose at admission was 0.76 µg/kg/min. Median ICU and total length of hospital stay were 6 and 7 days, respectively. One patient died within 12 months of admission, with this death occurring during the hospitalization period. A Kaplan–Meier survival plot is shown in Figure 1.

### 3.2. Urinary Cysteinyl Leukotrienes at Admission

**Figure 2:**
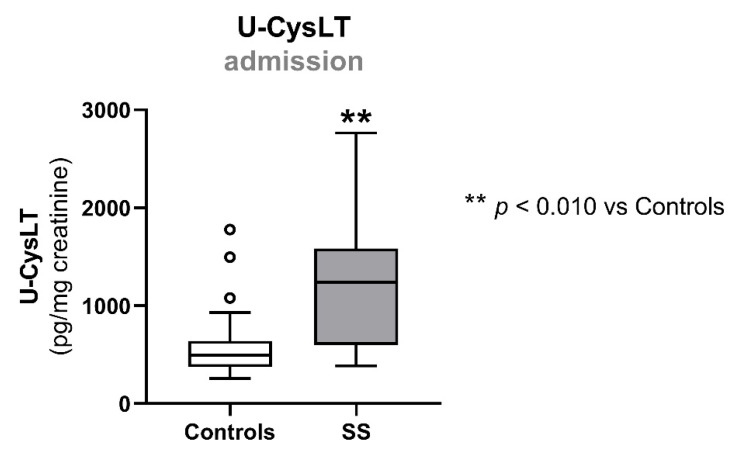
Admission values of u-CysLT in controls (*n* = 22) and SS patients (*n* = 13). Results are presented as box-and-whiskers plot. ** *p* < 0.010 vs. controls. SS, septic shock; u-CysLT, urinary cysteinyl leukotrienes.

SS patients had significantly higher concentrations of u-CysLT at admission (1242 (599; 1583) pg/mg creatinine vs. 495 (373; 639) pg/mg creatinine, *p* < 0.010, SS vs. controls) (Figure 2 and Table 2).

### 3.3. Biomarkers of Endothelial Activation, Inflammation, Oxidative Stress, and RAAS at Admission

At ICU admission, patients with SS showed significantly exacerbated endothelial activation, inflammation, and oxidative stress, as demonstrated by the markedly higher values of endothelial-adhesion molecules, inflammatory cytokines, MPO, and u-Isop (Table 2). Furthermore, they also presented markedly increased p-AGT and u-AGT values, suggesting enhanced activation of both systemic and intrarenal RAAS (Table 2).

### 3.4. Evolution of Urinary Cysteinyl Leukotrienes during Hospitalization

**Figure 3:**
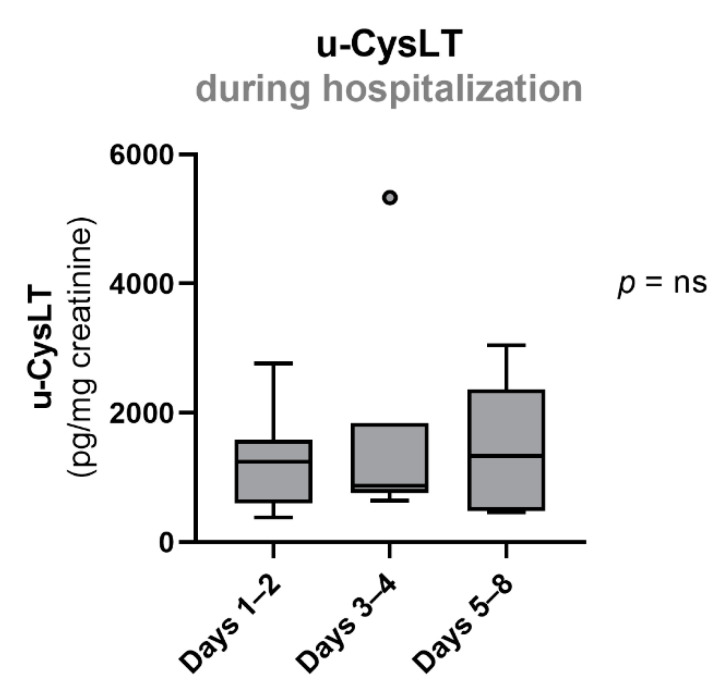
Evolution of u-CysLT in SS patients during hospitalization. Results are presented as box-and-whiskers plot, days 1–2 (*n* = 13), days 3–4 (*n* = 7), days 5–8 (*n* = 5); u-CysLT, urinary cysteinyl leukotrienes.

The values of u-CysLT in SS patients did not significantly change during hospitalization (u-CysLT (pg/mg creatinine), days 1–2: 1242 (599; 1583); days 3–4: 873 (758; 1842); days 5–8: 1332 (484; 2368)) (Figure 3 and Table 3).

### 3.5. Evolution of Endothelial Activation, Inflammation, Oxidative Stress, and RAAS during Hospitalization

There was a significant reduction in all endothelial-activation markers and in some inflammatory markers (s-TNF-α, s-IL-1β, s-CRP, NLR, and NMR) throughout hospitalization (Table 3), although the values of these biomarkers remained above control (Table 2) or normal reference values (normal CRP: <3 mg/L; normal NLR: 1–3). The values of u-Isop, which reflect oxidative-stress status, remained unchanged and elevated during hospitalization (Table 3). Regarding RAAS parameters, neither p-AGT nor u-AGT significantly changed throughout hospitalization (Table 3).

### 3.6. Evolution of Clinical and Biochemical Parameters of Organ Dysfunction during Hospitalization in SS Patients

SS patients had a significant reduction in lactate concentration and SOFA score and a significant improvement in eGFR throughout hospitalization. Urea was reduced at days 3–4, but at days 5–8 its values were not different from those at admission. Proteinuria remained unchanged and increased above normal values at days 3–4, but at days 5–8 there seemed to be a reduction, although not significant (Table 4). There was a reduction throughout hospitalization in hsTnI, a marker of cardiac injury, although this did not achieve statistical significance. Regarding respiratory parameters, there were no significant changes in P/F ratio during hospitalization, indicating the maintenance of acute hypoxemic respiratory failure (Table 4). SS patients did not exhibit an improvement in hepatic markers throughout hospitalization, with the exception of PT, which was significantly reduced (Table 4).

### 3.7. Correlation Analysis at Admission

**Figure 4:**
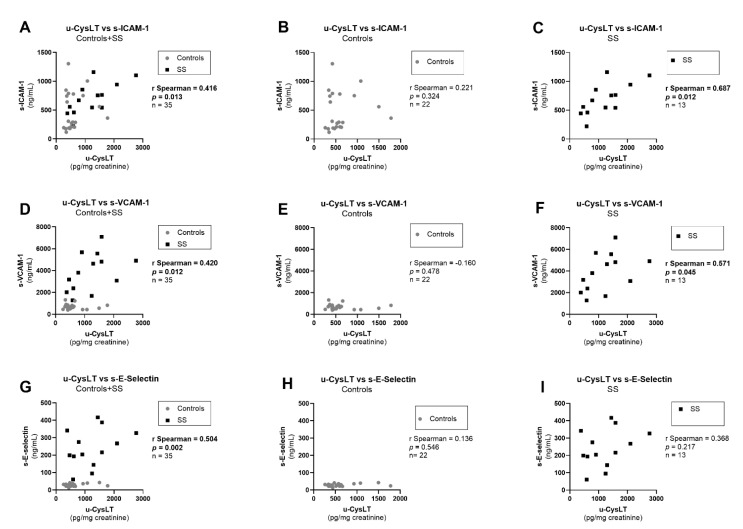
Correlations between u-CysLT and endothelial-activation biomarkers at admission in the overall study population and separately in controls and in SS: (**A**) u-CysLT vs. s-ICAM-1 in Controls + SS; (**B**) u-CysLT vs. s-ICAM-1 in Controls; (**C**) u-CysLT vs. s-ICAM-1 in SS; (**D**) u-CysLT vs. s-VCAM-1 in Controls + SS; (**E**) u-CysLT vs. s-VCAM-1 in Controls; (**F**) u-CysLT vs. s-VCAM-1 in SS; (**G**) u-CysLT vs. s-E-Selectin in Controls + SS; (**H**) u-CysLT vs. s-E-Selectin in Controls; (**I**) u-CysLT vs. s-E-Selectin in SS. s-ICAM-1, serum intercellular adhesion molecule 1; s-VCAM-1, serum vascular adhesion molecule 1; s-E-Selectin, serum E-Selectin; u-CysLT, urinary cysteinyl leukotrienes. The values of u-CysLT were corrected for the respective urinary creatinine concentrations.

We observed significant positive correlations of u-CysLT with endothelial-activation markers (u-CysLT vs. s-ICAM-1; u-CysLT vs. s-VCAM-1; u-CysLT vs. s-E-Selectin) at admission in the overall study population (Figure 4A,D,G). When analyzing controls and SS separately, significant positive correlations were observed in SS patients for u-CysLT vs. s-ICAM-1 (Figure 4C) and u-CysLT vs. s-VCAM-1 (Figure 4F), but not for u-CysLT vs. s-E-Selectin, although there seemed to be a positive correlation pattern in SS (Figure 4I). No significant correlation of u-CysLT with endothelial markers was found in controls alone (Figure 4B,E,H).

**Figure 5:**
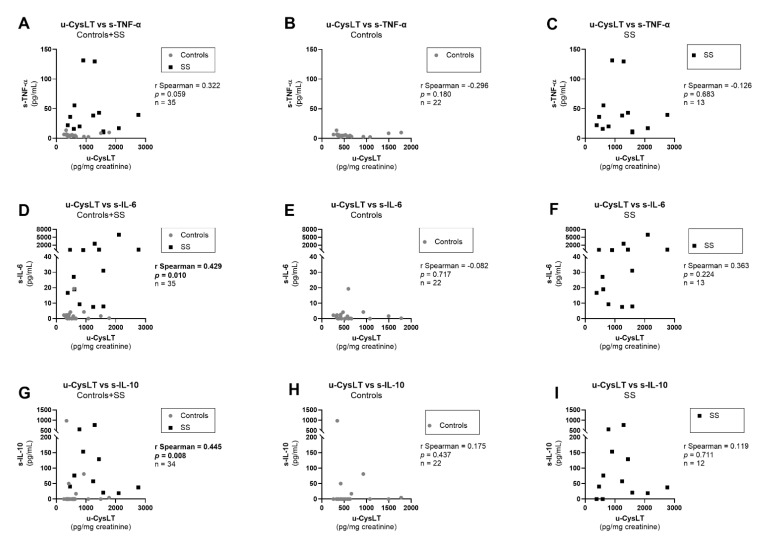
Correlations between u-CysLT and cytokines at admission in the overall study population and separately in controls and in SS: (**A**) u-CysLT vs. s-TNF-α in Controls + SS; (**B**) u-CysLT vs. s-TNF-α in Controls; (**C**) u-CysLT vs. s-TNF-α in SS; (**D**) u-CysLT vs. s-IL-6 in Controls + SS; (**E**) u-CysLT vs. s-IL-6 in Controls; (**F**) u-CysLT vs. s-IL-6 in SS; (**G**) u-CysLT vs. s-IL-10 in Controls + SS; (**H**) u-CysLT vs. s-IL-10 in Controls; (**I**) u-CysLT vs. s-IL-10 in SS. s-IL-6, serum interleukin 6; s-IL-10, serum interleukin 10; s-TNF-α, serum tumor necrosis factor alpha; u-CysLT, urinary cysteinyl leukotrienes. The values of u-CysLT were corrected for the respective urinary creatinine concentrations.

**Figure 6:**
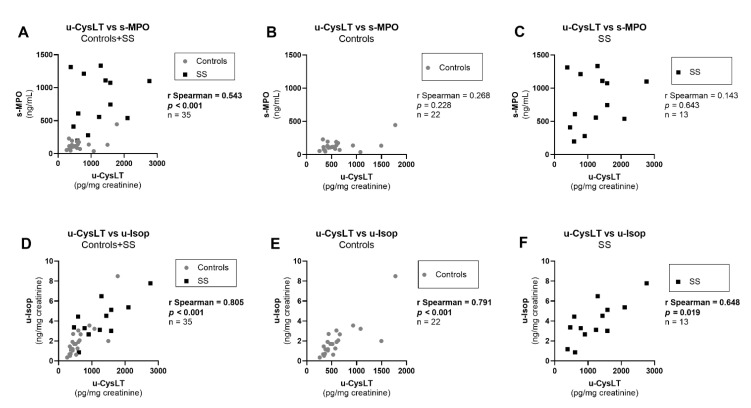
Correlation between u-CysLT and markers of inflammation and oxidative stress at admission in the overall study population and separately in controls and in SS: (**A**) u-CysLT vs. s-MPO in Controls + SS; (**B**) u-CysLT vs. s-MPO in Controls; (**C**) u-CysLT vs. s-MPO in SS; (**D**) u-CysLT vs. u-Isop in Controls + SS; (**E**) u-CysLT vs. u-Isop in Controls; (**F**) u-CysLT vs. u-Isop in SS. s-MPO, serum myeloperoxidase; u-CysLT, urinary cysteinyl leukotrienes; u-Isop, urinary isoprostanes. The values of u-CysLT and u-Isop were corrected for the respective urinary creatinine concentrations.

In the overall study population, u-CysLT were also significantly positively correlated with several inflammation and oxidative-stress markers (u-CysLT vs. s-IL-6; u-CysLT vs. s-IL-10; u-CysLT vs. s-MPO; u-CysLT vs. u-Isop) (Figure 5D,G and Figure 6A,D, respectively). Additionally, a borderline significant positive correlation was also found between u-CysLT and s-TNF-α (Figure 5A). However, when analyzing groups separately, u-CysLT only remained significantly positively correlated with u-Isop, both for controls and for SS (Figure 6E,F). No significant correlations were found in controls or in SS alone for u-CysLT vs. s-TNF-α, u-CysLT vs. s-IL-6, u-CysLT vs. s-IL-10, or u-CysLT vs. s-MPO (Figure 5B,C,E,F,I and Figure 6B,C, respectively). There were no significant correlations between u-CysLT and IL-1β in the overall study population (r Spearman = 0.143, *p* = 0.413, *n* = 35), in controls (r Spearman = −0.233, *p* = 0.296, *n* = 22), or in SS (r Spearman = 0.330, *p* = 0.296, *n* = 13).

**Figure 7:**
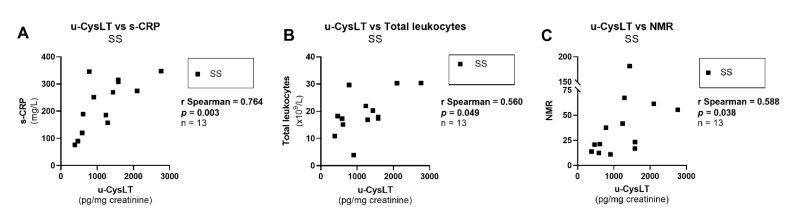
Correlations between u-CysLT and routine clinical inflammatory markers/parameters at admission in SS patients: (**A**) u-CysLT vs. s-CRP; (**B**) u-CysLT vs. total leukocytes; (**C**) u-CysLT vs. NMR; NMR, neutrophil-to-monocyte ratio; u-CysLT, urinary cysteinyl leukotrienes. The values of u-CysLT were corrected for the respective urinary creatinine concentrations.

The analysis of correlations of u-CysLT with routine clinical inflammatory biomarkers/parameters was conducted only in SS patients, revealing significant positive correlations for u-CysLT vs. s-CRP, u-CysLT vs. total leukocytes and u-CysLT vs. NMR; (Figure 7A–C), but not for u-CysLT vs. neutrophils (r Spearman = 0.473, *p* = 0.106, *n* = 13), u-CysLT vs. lymphocytes (r Spearman = −0.203, *p* = 0.505, *n* = 13), u-CysLT vs. monocytes (r Spearman = −0.369, *p* = 0.214, *n* = 13), and u-CysLT vs. NLR (r Spearman = 0.484, *p* = 0.097, *n* = 13).

**Figure 8:**
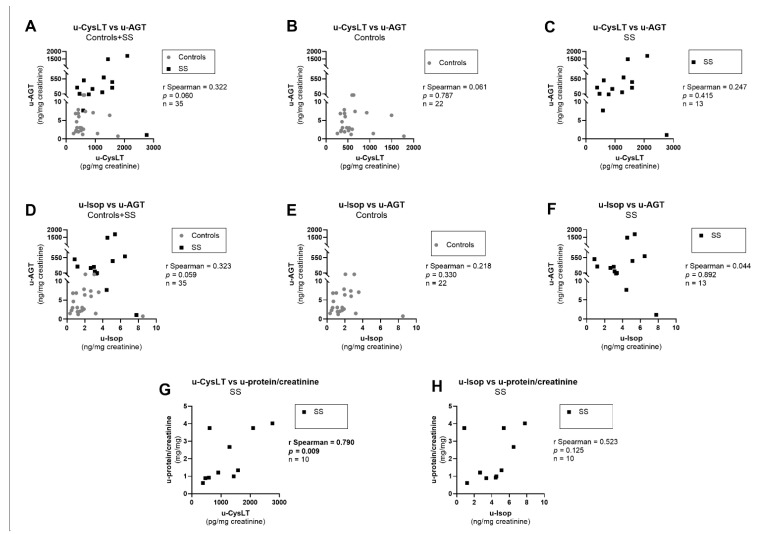
Correlations for u-CysLT or u-Isop with biomarkers of systemic/intra-renal RAAS and renal injury at admission: (**A**) u-CysLT vs. u-AGT in overall study population; (**B**) u-CysLT vs. u-AGT in Controls; (**C**) u-CysLT vs. u-AGT in SS; (**D**) u-Isop vs. u-AGT in overall study population; (**E**) u-Isop vs. u-AGT in Controls; (**F**) u-Isop vs. u-AGT in SS; (**G**) u-CysLT vs. u-protein/creatinine in SS; (**H**) u-Isop vs. u-protein/creatinine in SS. RAAS, renin–angiotensin–aldosterone system; u-AGT, urinary angiotensinogen; u-CysLT, urinary cysteinyl leukotrienes; u-Isop, urinary isoprostanes; u-protein/creatinine ratio, urinary protein/creatinine. The values of u-CysLT, u-Isop and u-AGT were corrected for the respective urinary creatinine concentrations.

Regarding RAAS and renal dysfunction markers, in the overall study population, admission values of u-CysLT showed a borderline positive correlation with u-AGT (Figure 8A), a marker of intrarenal RAAS activation, which in turn also had a borderline significant positive correlation with u-Isop (Figure 8D). When analyzing these correlations separately in controls and in SS, no significant correlations were found between u-CysLT vs. u-AGT (Figure 8B,C) or u-Isop vs. u-AGT (Figure 8E,F,) in controls or in SS. Nevertheless, there seemed to be a positive correlation pattern between u-CysLT and u-AGT in SS, as suggested by graphic analysis (Figure 8C). There were no significant correlations between u-CysLT and p-AGT (overall study population, r Spearman = 0.231, *p* = 0.182, *n* = 35; controls, r Spearman = −0.178, *p* = 0.428, *n* = 22; SS patients, r Spearman = 0.225, *p* = 0.459, *n* = 13), nor between u-Isop and p-AGT (overall study population, r Spearman = 0.123, *p* = 0.482, *n* = 35; controls, r Spearman = −0.185, *p* = 0.411, *n* = 22; SS patients, r Spearman = −0.049, *p* = 0.878, *n* = 13). Regarding data on proteinuria, analyzed only in SS patients, we observed that u-CysLT, but not u-Isop, were significantly positively correlated with u-protein/creatinine (Figure 8G,H).

**Figure 9:**
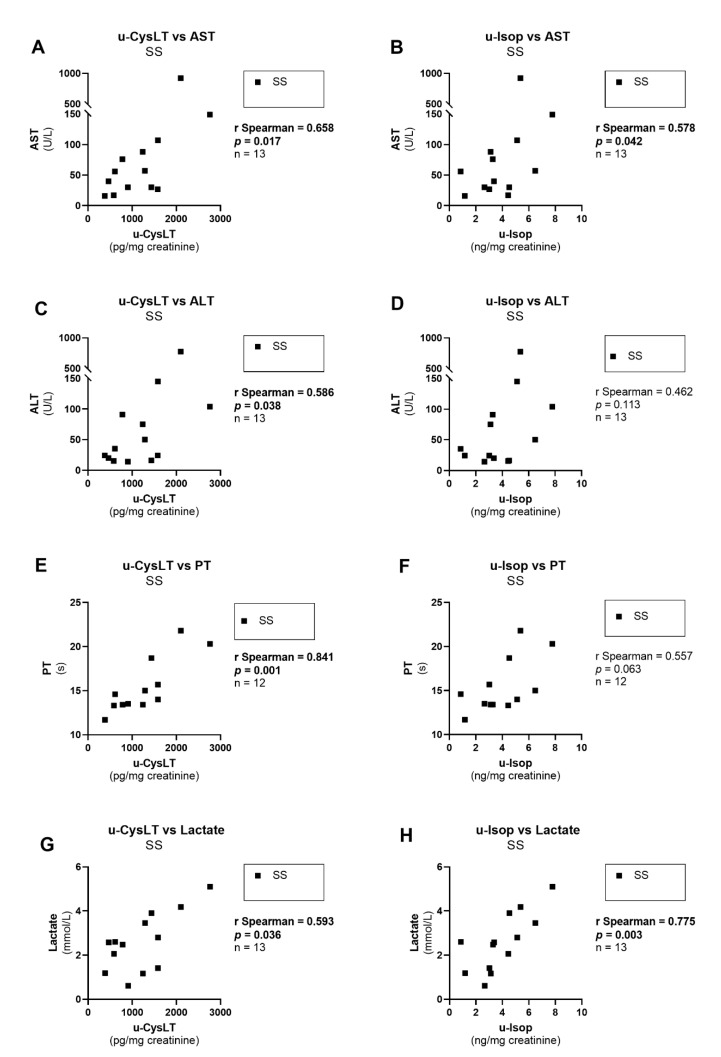
Correlations for u-CysLT or u-Isop with hepatic biomarkers and lactate at admission in SS patients: (**A**) u-CysLT vs. AST; (**B**) u-Isop vs. AST; (**C**) u-CysLT vs. ALT; (**D**) u-Isop vs. ALT; (**E**) u-CysLT vs. PT; (**F**) u-Isop vs. PT; (**G**) u-CysLT vs. lactate; (**H**) u-Isop vs. lactate. ALT, alanine aminotransferase; AST, aspartate aminotransferase; u-CysLT, urinary cysteinyl leukotrienes; u-Isop, urinary isoprostanes. The values of u-CysLT and u-Isop were corrected for the respective urinary creatinine concentrations.

With respect to the relation of u-CysLT with hepatic parameters, analyzed only in SS patients, we observed significant positive correlations between u-CysLT and markers of hepatic injury/dysfunction (u-CysLT vs. AST; u-CysLT vs. ALT; u-CysLT vs. PT) (Figure 9A,C,E). A similar profile was also evidenced for u-Isop in SS patients, namely, a significant positive correlation with hepatic enzymes (u-Isop vs. AST) (Figure 9B) and a borderline positive correlation with PT) (Figure 9F), but u-Isop values were not significantly correlated with ALT (Figure 9D). Regarding the relationship with prognostic markers or scores, both u-CysLT and u-Isop had significant positive correlations with lactate (Figure 9G,H) but no correlation was found with prognostic scores (u-CysLT vs. APACHE II: r Spearman = 0.077, *p* = 0.803, *n* = 13; u-CysLT vs. SAPS II: r Spearman = 0.141, *p* = 0.645, *n* = 13; u-Isop vs. APACHE II: r Spearman = 0.102, *p* = 0.740, *n* = 13; u-Isop vs. SAPS II: r Spearman = −0.050, *p* = 0.874, *n* = 13).

### 3.8. Repeated-Measure Multivariate Analyses in SS Patients throughout Hospitalization

Repeated-measure multivariate analyses were conducted to determine the relationship between u-CysLT (dependent variable) and several biomarkers (independent variables) throughout hospitalization (days 1–2, days 3–4, and days 5–8), adjusting for potential confounders, namely, age, gender, and eGFR. For each of the biomarkers, an independent model was developed.

We observed that the following biomarkers were positively associated with u-CysLT after adjusting to confounders: s-ICAM-1 (β = 1.760, *p* = 0.020); s-VCAM-1 (β = 0.268, *p* = 0.019); s-IL-6 (β = 0.219, *p* = 0.004); u-Isop (β = 299, *p* < 0.001); u-protein/creatinine (β = 434, *p* < 0.001); AST (β = 1.992, *p* < 0.001), and ALT (β = 1.523, *p* < 0.001). These repeated-measure multivariate analyses confirm the relationship between u-CysLT and endothelial activation markers (ICAM-1, VCAM-1), inflammatory (IL-6) and oxidative stress markers (u-Isop), renal injury (u-protein/creatinine), and hepatic markers (AST, ALT) previously detected in correlation analyses at admission, with higher values of these biomarkers being associated with higher u-CysLT values throughout hospitalization (Table 5).

## 4. Discussion

Our study highlights the importance of CysLT in the initiation and progression of sepsis cascade in SS patients, evidencing their correlation with major deleterious mechanisms contributing to multiple organ failure, such as inflammation, endothelial dysfunction, and oxidative stress.

CysLT have been shown to contribute to the pathophysiology of experimental sepsis [51,52,53,54,55]. However, their role in human sepsis or SS has scarcely been explored, with only one study evaluating their ex vivo production by leukocytes from critically ill patients with sepsis [46]. As far as we know, this is the first study assessing u-CysLT in SS patients. Of note, even experimental studies of sepsis have rarely measured CysLT in urine, with most reports describing their quantification in the plasma [51,58,59,60,61], although these mediators have a short half-life in blood circulation [39]. In the present study, we observed that SS patients had increased u-CysLT values at ICU admission [38,39]. Previous experimental studies have evidenced CysLT as mediators of LPS-induced shock. Indeed, CysLT receptor antagonists or LT synthesis inhibitors are protective in rodent models of sepsis or SS [52,53,54,55]. In addition, the administration of small amounts of CysLT to guinea pigs induces similar symptoms to those elicited by LPS, including shock-like reactions, widespread vascular leakage, and myocardial depression [51]. Importantly, LPS was shown to induce the systemic production of CysLT while inhibiting the hepatobiliary clearance of CysLT and their metabolites, which is the main route for CysLT elimination in all species [51,62], thus potentiating the effects of CysLT in sepsis [51]. Therefore, in our SS patients, the marked increase in u-CysLT may result from the increased CysLT production elicited by LPS (since most SS cases were related to Gram-negative agents), as well as from the inhibition of their hepatobiliary clearance, resulting in a diversion to renal excretion.

Looking over the evolution of u-CysLT from days 1–2 to days 5–8 of the ICU stay, no significant changes were found among SS patients, with u-CysLT remaining increased in all studied time points. This is in accordance with results in critically ill patients with multiple trauma, in which the urinary excretion of LTE_4_ was increased during the first 10 days after trauma when compared to healthy subjects, and even more elevated in patients who developed acute respiratory-distress syndrome (ARDS) [63]. In contrast to our findings, a previous study evaluating LTC_4_ over time in septic ICU patients observed that ex vivo LTC_4_ generation by stimulated leukocytes was lower in septic patients than in controls, but significantly increased after one week in surviving septic patients compared to sepsis non-survivors, in which LTC_4_ production remained lowered [46]. The authors speculated that these effects reflect the patient’s cellular-energy state and redox dysfunction, which affect the synthetic capacity of essential substrates and mediators [46]. However, these results may not represent the in vivo conditions, where transcellular synthesis of CysLTs also occurs [41].

Endothelial damage is considered to be a hallmark of SS [2], in which there is a phenotypic change in endothelial cells towards a proinflammatory, proadhesive, procoagulant, and prooxidant state [26,64]. The proadhesive phenotype, characterized by enhanced expression of adhesion molecules [26], appears to be related to multiple organ failure and hemodynamic compromise in septic patients. Amalakuhan et al. showed that increased plasma concentrations of VCAM-1 and ICAM-1 are associated with multiple organ failure [65]. In another study, Cummings et al. concluded that E-selectin values correlated with hemodynamic compromise and severity of organ failure [66]. In our study, we found positive correlations between u-CysLT and endothelial biomarkers in the overall study population. Furthermore, the positive correlations observed between u-CysLT and ICAM-1 or between u-CysLT and VCAM-1 appeared to be specific for SS patients, since no correlation was found in controls when data analysis was performed separately in each group. These results were confirmed in the repeated-measure multivariate analysis, which also evidenced a positive association between u-CysLTs and the endothelial markers ICAM-1 and VCAM-1 throughout hospitalization. Thus, CysLT appear to be critical mediators of endothelial damage in SS patients. Accordingly, in previous studies in human umbilical-vein endothelial cells, CysLT were shown to potentiate the TNF-α-induced expression of VCAM-1 and leukocyte attachment to endothelial cells via the CysLT_2_ receptor [67]. In addition, CysLT also promoted endothelial-cell contraction and barrier disruption via the CysLT_2_ receptor and endothelial-cell proliferation via the CysLT_1_ receptor [67].

Proinflammatory cytokines, such as IL-6 and TNF-α, seem to induce shock due to alterations in hematologic and hemodynamic status [68,69,70,71]. IL-6 is also a recognized endogenous pyrogen involved in multiple acute-phase responses, such as infection, tissue damage, and hepatic protein synthesis [72]. On the other hand, IL-10 is considered an anti-inflammatory cytokine with the ability to inhibit the production of proinflammatory cytokines, but also with immunosuppressive actions due to T-cell dysfunction and reduced antimicrobial function [73]. IL-10 concentrations can mirror the extent of the inflammatory status, with raised values being associated with adverse outcomes in sepsis [73]. In the overall study population, we found significant or borderline significant positive correlations of u-CysLT with these cytokines at admission. When analyzing data separately in each group, the pattern of positive correlation seemed to be maintained for IL-6 in SS patients, although not achieving statistical significance. Moreover, we also showed that u-CysLT were significantly positively correlated with s-CRP, s-MPO, total leukocytes, and NMR. These results reinforce the contribution of CysLT to the systemic inflammatory status found in a shock state. The correlations found probably reflect neutrophilic infiltration induced by CysLTs during sepsis since inflammatory immune cells such as leukocytes have an important role in the synthesis of CysLT [31,74], and IL-6 and TNF-α, which are also raised in our patients, increase the production and recruitment of polymorphonuclear leukocytes [75]. In addition, montelukast, a CysLT_1_ receptor antagonist, was already shown to inhibit MPO activity, a neutrophilic-infiltration marker, in the liver of LPS-treated rats [54], and higher MPO concentrations appear to be related to SS patients’ mortality [76]. 

Oxidative stress also plays an important role in sepsis pathophysiology. Indeed, oxidative stress and inflammation are closely interrelated. Proinflammatory cytokines induce the generation of ROS, which, in turn, activate several transcription factors that regulate the expression of proinflammatory cytokines [64,77]. Furthermore, activated inflammatory cells are known to produce large amounts of ROS [64]. Importantly, neutrophil-derived MPO can infiltrate into the vascular wall and use H_2_O_2_, produced by leukocyte oxidative burst and by vascular NADPH oxidases, to generate the potent prooxidant and proinflammatory hypochlorous acid, thus amplifying vascular injury under conditions of enhanced oxidative stress [78]. In addition, as previously mentioned, endothelial cells also contribute to a prooxidant environment during sepsis [64]. Ware et al. reported that multiple organ failure in critically ill septic patients was strongly associated with the serum concentration of isoprostanes [79]. In our study, u-Isop was markedly higher in SS patients and remained increased throughout hospitalization, being positively associated with u-CysLT. Treatment with montelukast, a CysLT_1_ receptor antagonist, was previously shown to prevent the rise of MPO activity, GSH depletion, and lipid peroxidation in the liver of septic rats, suggesting that neutrophilic infiltration into tissues is a major contributor to sepsis-induced oxidative stress [54,55,80]. In addition, since LTC_4_ synthesis requires conjugation with reduced glutathione (GSH), a major antioxidant defense, the increased production of CysLT during SS may also enhance oxidative stress by depleting GSH cellular stores [46,81]. 

SS has a well-established association with multiple organ failure [82], being the most important etiology for acute kidney injury (AKI) in an ICU context [83]. In our study, u-AGT, which has been claimed as an index of intrarenal RAAS activation and a biomarker of AKI [84,85,86,87], was markedly increased in SS patients, being significantly positively correlated with u-Isop in the overall study population and also showing a borderline positive correlation with u-CysLT. Of note, the values of u-CysLT and u-AGT in SS patients seemed to be the major contributors to this positive correlation. Given the above-mentioned relationship between u-CysLT and u-Isop, confirmed by correlation and repeated-measure multivariate analyses, we hypothesize that u-CysLT-associated redox dysfunction is involved in intrarenal RAAS activation, evidenced by raised u-AGT values throughout hospitalization. Noteworthy, u-AGT was also reported to be a potential prognostic tool to identify patients in risk of worse outcomes related to AKI of multiple etiologies, including sepsis-associated AKI [88]. Additionally, the positive correlation of u-CysLT with proteinuria found in our SS patients at admission, as well as their positive association in repeated-measure multivariate analysis during hospitalization, suggests that CysLT are major contributors to sepsis-induced renal injury. Accordingly, montelukast was shown to protect against sepsis-induced renal injury in rats through anti-inflammatory and antioxidant effects [53,55]. This is even more interesting if we consider that in our patients the relationship between u-CysLT and proteinuria was maintained after adjusting for eGFR, given the prevalence of renal dysfunction in SS patients.

Liver dysfunction, although not representing the most common form of sepsis-induced organ injury, is associated with high mortality when it culminates in hepatic failure [54,89,90]. Furthermore, albeit traditionally considered a late feature of sepsis illness, liver dysfunction has also been shown as an early-occurring event in sepsis [89]. In our SS patients, u-CysLT and u-Isop values presented similar positive-correlation profiles with hepatic transaminases and PT. Although frequently multifactorial, this could also indicate liver injury and dysfunction triggered by CysLT and oxidative stress. Indeed, in an experimental model of sepsis, montelukast was shown to protect against liver damage by suppressing the release of proinflammatory and prooxidant mediators [54,55].

Finally, and probably with major clinical interest, is the positive correlation of u-CysLT and u-Isop with arterial-blood gas lactate at admission. Lactate is widely used in the ICU because increased lactate levels are related to the presence and severity of organ dysfunction, being also a surrogate of circulatory dysfunction [91,92,93]. Promptly accessible, it provides a globally accepted biomarker to help clinicians to quickly triage critical patients since it has been shown that survival decreases with increasing initial lactate levels [94], as confirmed in a recent ARISE trial [95]. Although its predictive value has been recognized by the SEPSIS-3 consensus definition of shock [8], lactate-guided resuscitation in septic shock continues to be a debated issue [96], especially after the ANDROMEDA-SHOCK trial [97]. In fact, in sepsis, the inflammatory response is associated with increased glycolysis and impaired pyruvate dehydrogenase and, consequently, with greater lactate formation [98]. Additionally, just like glucose, lactate may also serve as a substrate, particularly in stress like sepsis, for the liver to generate glucose, the brain to produce ATP, the heart [99], and mitochondria [100]. On the other side of this compensatory beneficial response, lactate may also mirror anaerobic glycolysis in hypoperfused territories with severe microcirculatory dysfunction [101], sympathetic activation of aerobic glycolysis [102], impaired hepatic lactate clearance with hepatosplanchnic ischemia [103], and mitochondrial dysfunction limiting pyruvate metabolism [98]. Given their close interrelationship, both u-CysLT and u-Isop may reflect and accompany both these beneficial and deleterious pathophysiological pathways in sepsis. As in our study, biomarkers of oxidative stress like plasma F2-isoprostanes and isofurans were shown to be associated with renal, hepatic, and coagulation failure, but not with circulatory or pulmonary failure in severe sepsis, confirming nevertheless that lipid peroxidation is a prominent feature of septic multisystem organ failure [79]. Nevertheless, so far, no specific drug has been approved for therapeutic targeting of isoprostanes or oxidative stress in clinical settings. In contrast, there are already clinically approved drugs such as CysLT_1_ receptor antagonists (e.g., montelukast), which effectively block CysLT effects. Therefore, CysLT may function not only as biomarkers but also as therapeutic targets that can be non-invasively monitored throughout hospitalization.

Although multi-organ failure pathophysiology in sepsis is poorly understood [17], our work provides important clues into CysLT’s intimate involvement in the complex inflammatory response triggered by infection. Pro- and anti-inflammatory mediators, which were evidently raised in our SS patients and correlated with u-CysLT, coordinate leukocyte diapedesis and infection resolution but can likewise promote tissue damage. Additionally, in our patients, the increased PT, as well as its positive correlation with u-CysLT—which also exhibited several positive correlations with inflammation and endothelial markers—is suggestive of u-CysLT’s contribution to intravascular coagulation, which is also a documented promotor of organ injury in sepsis both via endotoxin, as in the case of the majority of our urosepsis patients, or via cytokines like TNF, through induction of tissue-factor expression on monocytes and activated endothelial cells [104]. Inflammation-induced dysfunction of the vascular endothelium, accompanied by cell death and loss of barrier integrity [105], contribute to vasodilation, increased vascular permeability, and septic cardiomyopathy typical in SS. CysLT are known to be important mediators of microvascular permeability [42,47,48,49]. The fact that our patients had raised values of u-CysLT and serum endothelial-activation markers, which were significantly positively correlated with each other, reinforces once again CysLT’s contribution to SS. Emergent identification and treatment of these patients is imperative in order to prevent the cumulative effects of vascular and cardiac dysfunction, which culminates in cellular hypoxia and death. However, during reperfusion, upon O_2_ reintroduction, there is an exacerbation of ROS release as a result of the metabolic adaptations and dysfunctional mitochondrial homeostasis induced by ischemia [106]. This aggravates oxidative stress, further damaging cells and causing organ injury and dysfunction [107]. The higher values of u-CysLT, u-Isop, and lactate in our patients, as well as the significant positive correlations found between these biomarkers, also evidence the important role of CysLT in SS pathophysiology. Although our work was not designed nor powered enough to evaluate the correlation of these biomarkers with prognosis, notably, a recent study in SS patients demonstrated an association between higher mortality and increased concentrations at baseline of IL-6, TNF, IL-10 urine isoprostane, and lactate [108]. We consider that these findings add value to our study since all these biomarkers were also raised at admission and correlated with CysLT in our patients.

It is important to note that during hospitalization, the values of endothelial and inflammatory biomarkers in our SS patients remained higher than control values, despite their significant amelioration. Furthermore, SS therapy did not seem to reduce u-CysLT, oxidative stress, or RAAS activation markers or to improve most hepatic parameters in the time frame evaluated in this study. The positive associations of u-CysLT with endothelial-activation markers (ICAM-1 and VCAM-1), inflammation (IL-6), oxidative stress (u-Isop), proteinuria, and hepatic parameters (AST, ALT) throughout hospitalization further reinforces the relationship between u-CysLT and these pathophysiological processes in SS.

The major strengths of our study include the evaluation of an extensive panel of biomarkers related to SS pathophysiological mechanisms, in parallel to the quantification of CysLT, a mediator widely evidenced to play a major role in experimental sepsis but scarcely explored in human sepsis. Limitations include the small number of patients and the possible selection bias due to a single-center design and predominance of urosepsis, which could have limited the demonstration of the association of CysLT with severity scores, given the low in-hospital mortality observed. Given the exploratory nature of this study, which is part of a larger project not designed to specifically detect differences in CysLT, the role of u-CysLT in human SS should be further evaluated in larger and more heterogeneous populations of SS patients.

## 5. Conclusions

Collectively, our results indicate that u-CysLT are markedly increased in patients with SS, being closely related to endothelial dysfunction, inflammation, oxidative stress, and organ dysfunction. Thus, they may be potential non-invasive biomarkers for monitoring the pathophysiological mechanisms underlying SS and their evolution in response to therapy. Furthermore, given this tight pathophysiological correlation observed, we hypothesize that a potential novel therapeutical approach targeting the LT pathway (for example, testing CysLT receptor antagonists) could be of benefit and should be explored in the management of SS patients.

## Figures and Tables

**Table 1:** Demographic, clinical, and biochemical characteristics at admission and outcomes of study population.

Demographic, Clinical, and Biochemical Parameters	Controls (*n* = 22)	SS patients (*n* = 13)
**Age** (Years)	56 ± 1	62 ± 4
**Gender:** men, *n* (%)	13 (59)	5 (38)
**Gender:** women, *n* (%)	9 (41)	8 (62)
**APACHE II score**	n/a	26 ± 2
**SAPS II score**	n/a	55 ± 4
**SOFA score** **VV-ECMO,** ***n*** **(%)**	n/an/a	9 (7; 12) 1 (8)
**Lactate** (mmol/L) normal: <2	n.d.	2.6 (1.3; 3.7)
**Source of sepsis**, ***n*****(%)**		
Respiratory tract infection	n/a	2 (15)
Renal and genitourinary tract infection	n/a	10 (77)
Skin and cutaneous tissue infection	n/a	1(8)
**Microbiology,** ***n*** **(%)**		
Gram-negative	n/a	7 (54)
Other	n/a	2 (15)
Unidentified	n/a	4 (31)
**Comorbidities,** ***n*** **(%)**		
Diabetes mellitus type 2	n.d.	3 (23)
Arterial hypertension	n.d.	5 (38)
Atrial fibrillation	n.d.	3 (23)
Dyslipidemia	n.d.	3 (23)
Cardiovascular disease	n.d.	1 (8)
Obesity	n.d.	2 (15)
Alcohol abuse	n.d.	1 (8)
**Cardiac biomarkers**		
BNP (pg/mL) normal: <100	n.d.	382 (315; 526)
hsTnI (ng/L) normal: <34 (men); <16 (women)	n.d.	295 (74; 2251)
**Renal function**		
eGFR (mL/min per 1.73 m^2^) normal: ≥90; mildly reduced: 60–89	77 ± 4	36 ± 6
Urea (mg/dL) normal: 10–50 mg/dL	n.d	93 ± 13
Urine protein/creatinine (mg/mg) normal: <0.2	n.d.	2.0 ± 0.4
**Respiratory parameters**		
PaO_2_/FiO_2_ (P/F) ratio normal: ≥300	n/a	288 ± 31
**Hepatic biomarkers**		
AST (U/L) normal: 10–37	n.d.	56 (29; 98)
ALT (U/L) normal: 10–37	n.d.	35 (18; 98)
ALP (U/L) normal: 30–120	n.d.	69 (65; 106)
GGT (U/L) normal: 10–49	n.d.	37 (19;68)
Total bilirubin (mg/dL) normal: <1.2	n.d.	0.9 (0.5; 3.1)
PT (s) normal: 9.6–13.6	n.d.	14.3 (13.4; 18.0)
**Therapeutics at admission**		
Norepinephrine dose (µg/kg/min)	n/a	0.76 ± 0.15
Use of renal replacement therapy, *n* (%)	n/a	2 (15)
Antibiotic therapy, *n* (%)		
Ceftriaxone	n/a	9 (69)
Piperacillin/tazobactam	n/a	4 (31)
Dual or triple combination	n/a	4 (31)
Antiviral therapy, *n* (%)	n/a	1 (8)
**Outcomes**		
ICU length of stay (days)	n/a	6 (4; 9)
Total hospital length of stay (days)	n/a	7 (5; 11)
In-hospital mortality, *n* (%)	n/a	1 (8)
Mortality within 12 months, *n* (%)	n/a	1 (8)

ALP, alkaline phosphatase; ALT, alanine aminotransferase; APACHE, acute physiology and chronic health evaluation; ARDS, acute respiratory-distress syndrome; AST, aspartate aminotransferase; BNP, B-type natriuretic peptide; eGFR, estimated glomerular filtration rate; FiO_2_, fraction of inspired oxygen; GGT, gamma glutamyltransferase; hsTnI, high-sensitivity troponin I; ICU, intensive care unit; n/a, not applicable; n.d., not determined; PaO_2_, partial pressure of arterial oxygen; P/F, PaO_2_/FiO_2_ ratio; PT, prothrombin time; SAPS, Simplified Acute Physiology Score; SOFA, Sequential Organ Failure Assessment; VV-ECMO, veno-venous extracorporeal membrane oxygenation. Results are expressed as number (%), mean ± SEM, or median (25th percentile; 75th percentile) for data with normal or non-normal distribution, respectively.

**Table 2:** Admission values of biomarkers of endothelial activation, inflammation, oxidative stress, and RAAS in controls and SS patients.

Biomarkers	Controls	*n*	SS	*n*	*p*-Value
**Endothelial activation**					
s-ICAM-1 (ng/mL)	474.9 ± 70.7	22	694.5 ± 75.3	13	0.038
s-VCAM-1 (ng/mL)	705.1 (516.2; 827.7)	22	3810.0 (2201.0; 5232.0)	13	<0.001
s-E-Selectin (ng/mL)	27.7 (22.6; 34.1)	22	216.4 (169.1; 334.5)	13	<0.001
**Inflammatory status**					
s-TNF-α (pg/mL)	4.8 (3.6; 6.2)	22	36.3 (16.6; 49.3)	13	<0.001
s-IL-1β (pg/mL)	0.0 (0.0; 0.2)	22	0.2 (0.1; 0.5)	13	0.010
s-IL-6 (pg/mL)	0.7 (0.0; 2.4)	22	31.0 (12.9; 293.2)	13	<0.001
s-IL-10 (pg/mL)	0.0 (0.0; 1.2)	22	49.1 (19.5; 147.6)	12	<0.001
s-MPO (ng/mL)	116 (76; 149)	22	746 (475; 1162)	13	<0.001
s-CRP (mg/L)	n.d.	-	225.3 ± 26.4	13	n/a
Total leukocytes (×10^9^/L)	n.d.	-	19.2 ± 2.1	13	n/a
Neutrophils (×10^9^/L)	n.d.	-	17.1 ± 1.9	13	n/a
Monocytes (×10^9^/L)	n.d.	-	0.6 ± 0.1	13	n/a
Lymphocytes (×10^9^/L)	n.d.	-	0.6 (0.5; 0.8)	13	n/a
NLR	n.d.	-	23.3 (14.2; 45.7)	13	n/a
NMR	n.d.	-	23.3 (15.3; 58.4)	13	n/a
u-CysLT (pg/mg creatinine)	495 (373; 639)	22	1242 (599; 1583)	13	0.003
**Oxidative stress**					
u-Isop (ng/mg creatinine)	1.7 (1.0; 2.7)	22	3.4 (2.8; 5.2)	13	0.002
**Systemic and intrarenal RAAS**					
p-AGT (μg/mL)	19.6 (17.5; 24.4)	22	35.3 (21.4; 46.4)	13	0.003
u-AGT (ng/mg creatinine)	3 (2; 7)	22	256 (45; 549)	13	<0.001

n/a, not applicable; n.d., not determined; NLR, neutrophil-to-lymphocyte ratio; NMR, neutrophil-to-monocyte ratio; p-AGT, plasma angiotensinogen; RAAS, renin–angiotensin–aldosterone system; s-CRP, serum C-reactive protein; s-ICAM-1, serum intercellular-adhesion molecule 1; s-IL-1β, serum interleukin 1 beta; s-IL-6, serum interleukin 6; s-IL-10, serum interleukin 10; s-MPO, serum myeloperoxidase; s-TNF-α, serum tumor necrosis factor alpha; s-VCAM-1, serum vascular cell-adhesion molecule 1; u-AGT, urinary angiotensinogen; u-Isop, urinary isoprostanes; u-CysLT, urinary cysteinyl leukotrienes. Results are expressed as mean ± SEM or as median (25th percentile; 75th percentile) for data with normal or non-normal distribution, respectively. The values of u-CysLT, u-Isop, and u-AGT were corrected for the respective urinary creatinine concentrations.

**Table 3:** Evolution of biomarkers of endothelial activation, inflammatory status, oxidative stress, and systemic and intrarenal RAAS during hospitalization in SS patients.

Biomarkers/Parameters	Days 1–2	*n*	Days 3–4	*n*	Days 5–8	*n*
**Endothelial activation**						
s-ICAM-1 (ng/mL)	694.5 ± 75.3	13	559.0 ± 92.1 *	8	553.3 ± 85.3	6
s-VCAM-1 (ng/mL)	3855.0 ± 488.7	13	2559.0 ± 479.3	8	1912.0 ± 488.8 *	6
s-E-Selectin (ng/mL)	240.9 ± 30.1	13	137.1 ± 23.2 **	8	138.2 ± 24.1 **	6
**Inflammatory status**						
s-TNF-α (pg/mL)	36.3 (16.6; 49.3)	13	13.3 (5.9; 18.9) *	8	14.2 (8.9; 38.8)	6
s-IL-1β (pg/mL)	0.21 (0.07; 0.48)	13	0.03 (0.01; 0.08) *	8	0.15 (0.00; 0.68)	6
s-IL-6 (pg/mL)	31.0 (12.9; 293.2)	13	23.2 (5.6; 54.4)	8	11.4 (6.8; 25.3)	6
s-IL-10 (pg/mL)	49.1 (19.5; 147.6)	12	18.5 (1.7; 70.9)	8	5.4 (2.4; 39.7)	5
s-MPO (mg/mL)	746 (475; 1162)	13	548 (301; 688)	8	227 (192; 590)	6
s-CRP (mg/L)	225.3 ± 26.4	13	162.0 ± 43.6*	8	91.5 ± 19.2 *	5
Total leukocytes (×10^9^/L)	19.2 ± 2.1	13	18.0 ± 3.7	8	12.1 ± 3.8	6
Neutrophils (×10^9^/L)	17.1 ± 1.9	13	14.8 ± 3.4	8	8.8 ± 2.8	6
Monocytes (×10^9^/L)	0.6 ± 0.1	13	0.8 ± 0.1	8	0.9 ± 0.2	6
Lymphocytes (×10^9^/L)	0.6 (0.5; 0.8)	13	1.3 (0.9; 2.2)	8	1.3 (0.9; 1.8)	6
NLR	23.3 (14.2; 45.7)	13	7.3 (5.8; 18.7) **	8	6.6 (3.4; 8.6) *	6
NMR	23.3 (15.3; 58.4)	13	19.10 (8.4; 29.6) **	8	9.9 (5.4; 13.5) *	6
u-CysLT (ng/mg creatinine)	1242 (599; 1583)	13	873 (758; 1842)	7	1332 (484; 2368)	5
**Oxidative stress**						
u-Isop (ng/mg creatinine)	3.4 (2.8; 5.2)	13	4.2 (2.7; 10.1)	7	3.6 (2.2; 7.7)	5
**Systemic and intrarenal RAAS**						
p-AGT (μg/mL)	35.4 ± 4.1	13	36.5 ± 4.8	8	36.6 ± 6.8	6
u-AGT (ng/mg creatinine)	256 (45; 549)	13	104 (57; 361)	8	316 (143; 1735)	6

NLR, neutrophil-to-lymphocyte ratio; NMR, neutrophil-to-monocyte ratio; p-AGT, plasma angiotensinogen; RAAS, renin–angiotensin–aldosterone system; s-CRP, serum C-reactive protein; s-ICAM-1, serum intercellular-adhesion molecule 1; s-IL-1β, serum interleukin 1 beta; s-IL-6, serum interleukin 6; s-IL-10, serum interleukin 10; s-MPO, serum myeloperoxidase; s-TNF-α, serum tumor necrosis factor alpha; s-VCAM-1, serum vascular cell-adhesion molecule 1; u-AGT, urinary angiotensinogen; u-Isop, urinary isoprostanes; u-CysLT, urinary cysteinyl leukotrienes. Results are expressed as mean ± SEM or as median (25th percentile; 75th percentile) for data with normal or non-normal distribution, respectively. * *p* < 0.05 vs. days 1–2; ** *p* < 0.01 vs. days 1–2. The values of u-CysLT, u-Isop, and u-AGT were corrected for the respective urinary creatinine concentrations.

**Table 4:** Clinical and biochemical parameters of organ dysfunction during hospitalization in SS patients.

Parameters	Days 1–2	*n*	Days 3–4	*n*	Days 5–8	*n*
**Lactate** (mmol/L)	2.6 (1.3; 3.7)	13	1.3 (1.1; 1.6) *	8	1.5 (1.1; 1.7)	4
**SOFA score**	9 (7; 12)	13	6 (2; 11)	8	6 (4; 7) *	4
**Cardiac biomarkers**						
BNP (pg/mL)	382 (315; 526)	8	n.d.	-	n.d.	-
hsTnI (ng/L)	295 (74; 2251)	13	128 (32; 5460)	6	10 (5; 495)	4
**Renal function**						
eGFR (mL/min per 1.73 m^2^)	36 ± 6	13	62 ± 12 *	8	82 ± 12 **	5
Urea (mg/dL)	79 (59; 124)	13	55 (44; 82) *	8	71 (46; 125)	6
Urine protein/creatinine (mg/mg)	2.0 ± 0.4	10	2.0 ± 0.5	6	1.0 ± 0.3	3
**Respiratory parameters**						
PaO_2_/FiO_2_ (P/F) ratio	288 ± 31	12	282 ± 43	8	223 ± 12	5
**Hepatic biomarkers**						
AST (U/L)	56 (29; 98)	13	61 (17; 236)	6	86 (41; 476)	5
ALT (U/L)	35 (18; 98)	13	90 (42; 309)	6	119 (49; 454)	5
ALP (U/L)	69 (65; 106)	13	125 (92; 214) *	6	227 (108; 297)	5
GGT (U/L)	37 (19;68)	13	81 (47; 89) *	6	135 (77; 217)	4
Total bilirubin (mg/dL)	0.9 (0.5; 3.1)	13	0.9 (0.5; 3.6)	6	1.9 (0.6; 8.8)	5
PT (s)	14.3 (13.4; 18.0)	12	13.6 (12.7; 14.5) *	8	12.9 (11.0; 15.2)	5

ALP, alkaline phosphatase; ALT, alanine aminotransferase; AST, aspartate aminotransferase; BNP, B-type natriuretic peptide; eGFR, estimated glomerular filtration rate; FiO_2_, fraction of inspired oxygen; GGT, gamma glutamyltransferase; hsTnI, high-sensitivity troponin I; n.d., not determined; PaO_2_, partial pressure of arterial oxygen; P/F, PaO_2_/FiO_2_ ratio; PT, prothrombin time; SOFA, Sequential Organ Failure Assessment. Results are expressed as mean ± SEM or as median (25th percentile; 75th percentile) for data with normal or non-normal distribution, respectively. * *p* < 0.05 vs. days 1–2; ** *p* < 0.01 vs. days 1–2.

**Table 5:** Repeated-measure multivariate models for u-CysLT in SS patients throughout hospitalization. Adjusted β, 95% confidence intervals (95% CI), and *p*-value estimated by repeated-measure multivariate models with u-CysLT as the dependent variable and adjusted for age, gender, and eGFR.

u-CysLT (pg/mg Creatinine)	Adjusted β	95% CI	*p*-Value
Model 1			
**s-ICAM-1** (ng/mL)	1.760	0.281–3.238	0.020
Model 2			
**s-VCAM-1** (ng/mL)	0.268	0.043–0.492	0.019
Model 3			
**s-IL-6** (pg/mL)	0.219	0.068–0.369	0.004
Model 4			
**u-Isop** (ng/mg creatinine)	299	185–414	<0.001
Model 5			
**u-protein/creatinine** (mg/mg)	434	226–642	<0.001
Model 6			
**AST** (U/L)	1.992	0.936–3.048	<0.001
Model 7			
**ALT** (U/L)	1.523	0.719–2.326	<0.001

AST, aspartate aminotransferase; ALT, alanine aminotransferase; s-ICAM-1, serum intercellular-adhesion molecule 1; s-IL-6, serum interleukin 6; s-VCAM-1, serum vascular-adhesion molecule 1; u-CysLT, urinary cysteinyl leukotrienes; u-Isop, urinary isoprostanes.

## Data Availability

The datasets used and/or analyzed during the current study are available from the corresponding author on reasonable request.
